# *In vitro* and *in vivo* proves of concept for the use of a chemically cross-linked poly(ester-urethane-urea) scaffold as an easy handling elastomeric biomaterial for bone regeneration

**DOI:** 10.1093/rb/rbz020

**Published:** 2019-04-23

**Authors:** Géraldine Rohman, Sylvie Changotade, Sophie Frasca, Salah Ramtani, Anne Consalus, Credson Langueh, Jean-Marc Collombet, Didier Lutomski

**Affiliations:** 1 Université Paris 13, Sorbonne Paris Cité, Tissue Engineering and Proteomics (TIP) Team, CSPBAT, UMR CNRS 7244, 74 rue Marcel Cachin, 93000 Bobigny, France; 2 Département Soutien Médico-Chirurgical des Forces (SMCF), BP73, Institut de Recherche Biomédicale des Armées (IRBA), 91223 Brétigny-sur-Orge Cedex, France; 3 Université Paris 13, Sorbonne Paris Cité, LBPS Team, CSPBAT, UMR CNRS 7244, 99 Avenue Jean-Baptiste Clément, 93430 Villetaneuse, France

**Keywords:** bone regeneration, scaffold, elastomer, PEUU, PCLU

## Abstract

Bone loss can occur as a result of various pathologies, traumas and injuries and poor bone healing leads to functionally debilitating condition, loss of self-sufficiency and deterioration in life quality. Given the increasing incidence of facial trauma and the emergence of new procedural techniques, advanced scaffolds are currently developed as substitutes for bone tissue engineering. In this study, we investigated the capability of a chemically cross-linked ε-caprolactone-based poly(ester-urethane-urea) (PCLU) scaffold to support bone regeneration. *In vitro* assays demonstrated that PCLU scaffolds could be colonized by cells through direct cell seeding and cell migration from outside to scaffold inside. Moreover, PCLU scaffolds could provide a suitable environment for stem cells proliferation in a 3D spatial arrangement, and allowed osteogenic differentiation under appropriate induction. *In vivo* results revealed the osteogenic properties of PCLU scaffolds through a drilled-hole femoral bone defect repair improvement in rats. Using histology and microtomography analysis, we showed that PCLU scaffolds fit well the bone cavity and were eventually entrapped between the newly formed trabeculae. Finally, no sign of inflammation or rejection was noticed. We envision that PCLU scaffolds can provide the clinicians with a substitute having appropriate characteristics for the treatment of bone defects.

## Introduction

Most maxillary-craniofacial and long bone defects are the result of congenital deformities, diseases (bone loss after tumor resection), traumas (injuries caused by car, work or sport accidents) as well as war injuries (gunshots or explosive devices) [1]. When the defect site does not exceed a critical size, bone can spontaneously regenerate. For larger defect area, reconstructive surgery must be considered for successful bone repair. To date, the ‘gold standard’ surgery procedure is an autologous graft using the own patient bone tissue collected from the iliac crest or one of the patient intact long bones by the reamer-irrigator aspirator (RIA) technique [2]. However, this autograft surgery is associated to several drawbacks, such as possible donor site infection, graft morbidity and limited available quantity of graft material. Furthermore, autografts are difficult to shape and settle inside the defect leading to unpredictable resorption due to a poor fitting [1, 3]. In some cases, despite reconstructive autograft surgery, only a partial osseous consolidation is achieved leading to non-union bone fractures (pseudarthrosis). Thus, the repair of large bone defect in both civilian and military populations remains a major challenge for orthopedic surgeons.

Current investigations aim at developing innovative bone substitutes that can provide reliable bone regeneration leading to functional and esthetic restoration. The ideal bone graft substitute should be biocompatible, bioresorbable, osteoconductive and osteoinductive. It should not present any risk of infectious disease contamination, be easy to use and readily available, as well as cost effective. A multitude of bone grafting materials are currently available on the market, including allografts, xenografts and alloplastic materials [4]. If autograft material is limited in quantity, allografts and xenografts are in turn available in abundance, but they may lead to disease transmission and immune rejection. Further processing can lower the risk, nevertheless the material osteoinductivity and mechanical properties are weakened [1, 3]. The most successful synthetic bone grafts currently available on the market are bioactive ceramics, such as tricalcium phosphate and hydroxyapatite. Indeed, their suitable biocompatibility, osteoconductivity and mechanical properties make them attractive compounds for bone tissue regeneration [5]. However, they exhibit well identified drawbacks, such as brittleness, uneasy shaping ability and poor bone defect fitting capacity leading to weak material–bone contact, thus, potentially inducing resorption risk of the new bone formed at the interface. Moreover, due to the lack of control over the bone graft degradation process, it is difficult to get bone repair and biomaterial resorption time-courses to concur [4, 6].

Nowadays, the increase in facial trauma incidence, the emergence of new procedural techniques and the better understanding of bone-healing biology, urge the need to optimize biomaterials for bone defect care, and leads to the development of advanced scaffolds for bone tissue engineering. In this strategy, the challenging goal is to elaborate scaffolds allowing cell adhesion and colonization, as well as vascularization and bone maturation. Indeed, the diamond concept suggests that three parameters are mandatory for successful bone regeneration: osteoconductive scaffold, osteoinductive growth factors and osteogenic cells [7]. In particular, the scaffold has to be designed with morphologies that fit the anatomic geometry of the defect, allowing to correctly define and maintain the space for the tissue regeneration, and with multiscale interconnected porous structure enabling cell migration, provision of oxygen and nutrients to engineered tissue prior to vascularization, as well as evacuation of metabolic wastes [8, 9]. Despite the wide variety of scaffolds found in the literature, based on biodegradable natural, synthetic and composite materials, a small number of studies addressed the combination of the three diamond concept elements since multicomponent approaches involved many questions and drawbacks that remain to be solved. [10–12]. Due to their large flexibility in terms of design, chemical composition, shape and physical properties, various synthetic polymers have been proposed as bone scaffold matrix. Among available biocompatible and biodegradable thermoplastic polymers, α-hydroxy polyesters are the most popular and widely used. They are approved by the FDA for clinical use, their physicochemical properties can be tailored and their degradation products are nontoxic [13]. However, their main disadvantages are the detrimental tissue response that may occurs when using these semicrystalline materials, and the surface chemical properties that need to be modified to enhance cell response [8]. Various hydrogels have been tested as promising candidates to obtain scaffold with a geometry that can perfectly match irregular bone defect outlines. Nevertheless, they often exhibit poor adhesion to the defect cavity borders, lack of mechanical strength, low stability for physical gels or *in vivo* adverse responses during chemical gel curing. Furthermore, scaffold cellular colonization can be limited by the hydrogel degradation rate due to the lack of adequate pore size and interconnected porosity [14–16]. A few studies have demonstrated the potentiality of elastomeric materials in bone tissue engineering. Indeed, their rubber-like elasticity would allow a convenient fitting into the bone defect, thus establishing an intimate contact with the native bone and providing a protection against shear forces at the bone–material interface. As a consequence, it was suggested that elastomeric scaffolds tend to facilitate the migration of osteogenic cells from native bone into the scaffold, and therefore promote bone regeneration [17–21]. In particular, polyurethane (PU), poly(ester-urethane) (PEU) and poly(ester-urethane-urea) (PEUU) are of much interest since they are used in a wide range of biomedical applications, and can be synthesized with various chemical and mechanical properties, as well as with adjustable degradation rates [22]. Moreover, they are prone to favor bone mineralization which is a desired feature of bone graft substitutes [23]. Therefore, PU-based elastomers are promising biomaterials to support bone regeneration.

In a previous study, we assessed the *in vitro* compatibility of human mesenchymal stem cells (hMSC) toward a chemically cross-linked ε-caprolactone-based poly(ester-urethane-urea) (PCLU) scaffold [24]. PCLU scaffolds were developed by a simple and controlled manufacturing process using an emulsion technique named poly(HIPE) (high internal phase emulsion polymerized). This technique allows to elaborate scaffolds with various sizes and shapes combined with a multiscale and interconnected porosity. Since the porosity and pore sizes can be tailored by varying the emulsion parameters, it is therefore possible to easily develop scaffolds with adequate features for bone tissue engineering. We also demonstrated, in accordance with safety guideline standards, that PCLU scaffolds did not induce any release of cytotoxic by-products, exhibited a lack of cytotoxic response, and allowed hMSC adhesion and spreading over the pore walls after 7 days of culture. The present work aims at providing evidences that PCLU scaffolds are suitable biomaterials for supporting both *in vitro* and *in vivo* bone regeneration. For this purpose, *in vitro* studies were carried out to prove the potential of PCLU scaffolds to be colonized by various cells using direct static or dynamic seeding, as well as from outside to inside cell migration; to act as three-dimensional (3D) frameworks for cell proliferation; and to allow osteogenic differentiation of hMSC. At last, the *in vivo* osteogenic properties of PCLU scaffolds were also assessed in a rat femoral bone defect model, providing a standardized environment for studies of induction and remodeling of new bone.

## Materials and methods

### Materials

All solvents were purchased from Fisher and used as received. Phosphate-buffered saline (PBS) solution, Dulbecco’s modified Eagle’s medium (DMEM), fungizone antimycotic (Fz), penicillin (Pen), streptomycin (Strep) and trypsin-EDTA were supplied by Gibco Life Technologies. Poly(ε-caprolactone) oligomers, hexamethylene diisocyanate, Span^®^ 80, dibutyltin dilaurate, collagenase, paraformaldehyde (PFA), 2-phospho-l-ascorbic acid, β-glycerophosphate, DAPI, alizarin red, Triton X100 and cetylpyridinium chloride were purchased from Sigma-Aldrich. Fetal bovine serum (FBS) was obtained from PANBiotech GmbH.

Human dermal fibroblasts were isolated, using standard procedures, from foreskins of 4-year-old children with informed consent of the parents. Fibroblasts were used for the experiments between passages 4 and 6. hMSC were obtained by plastic adhesion from bone marrow samples collected from hematologically healthy patients undergoing routine total hip replacement surgery. All samples were obtained after informed consent from donors from the Hôpital d’Instruction des Armées Percy (Clamart, France).

### Scaffold elaboration and characterization

PCLU scaffolds were obtained through a poly-HIPE method and characterized by determination of their density, porosity and pore interconnectivity, as described previously [24]. The chemical composition of PCLU scaffolds was monitored by Fourier-transformed infrared spectroscopy (FTIR Nicolet 380—Thermo Scientific) in an attenuated total reflectance mode (ATR—smart omni sampler) within the range 500–4000 cm^−1^ with a resolution of 4 cm^−1^. The scaffold hydrophilicity/hydrophobicity was determined through water contact angle (WCA) measurement using a Digidrop Model DS GBX apparatus and Windrop++ software. The scaffold morphology was monitored using an environmental scanning electron microscope (ESEM TM3000—Hitachi) and by microcomputed tomography (micro-CT Quantum FX—Perkin Elmer). The scaffold elemental composition was determined by energy dispersive X-ray spectrometry using ESEM equipped with an EDX probe (Hitachi SwiftED3000).

The number average molecular weight between cross-links ${\bar{M}}_{c}$ of PCLU scaffolds was determined through swelling measurement. To do so, PCLU scaffolds were immersed in toluene at room temperature up to swelling equilibrium. Then, they were wiped and weighted to obtain the scaffold wet mass (*m*_wet_). ${\bar{M}}_{c}$ was calculated using the Flory-Rehner equation (1) [25]:

(1) $${\overset{-}{M}}_{c}=-\;\frac{V_{s}\times \left(\upsilon_{2s}^{1/3}-\;\frac{\upsilon_{2s}}{2}\right)}{\overset{-}{\upsilon }\times \left[\ln\left(1-\upsilon_{2s}\right)+\upsilon_{2s}+\chi \times \upsilon_{2s}^{2}\right]}$$

where *V*_s_ is the molar volume of the swelling agent (toluene), $\bar{\upsilon }$ is the specific volume of the polymer, *χ* is the Flory polymer–solvent interaction parameter calculated from the solubility parameters using Bristow and Watson equation (2) [26], *υ*_2s_ is the actual polymer volume fraction determined by Equation (3) [25].

(2) $$\chi =0.34+\frac{V_{s}}{\mathrm{RT}}\times {\left(\delta_{s}-\delta_{p}\right)}^{2}$$

(3) $$\frac{1}{\upsilon_{2s}}=\left[\frac{m_{\mathrm{dry}}\times \rho_{\mathrm{nonporous}}\times \left(\frac{\rho_{s}}{\rho_{\mathrm{porous}}}\;-1\right)+m_{\mathrm{wet}}\times \rho_{\mathrm{nonporous}}}{m_{\mathrm{dry}}\times \rho_{s}}\right]-\left[\frac{P}{1-P}\right]$$

In Equation (2), *V*_s_ is the molar volume of the solvent (toluene), *R* is the universal gas constant, *T* is the absolute temperature, *δ*_s_ is the solubility parameter of the solvent (toluene) and *δ*_p_ is the solubility parameter of the polymer network calculated using the group contribution method based on Van Krevelen’s molar attraction constants [27]. In Equation (3), *m*_dry_ is the mass of the scaffold before swelling, *m*_wet_ is the mass of the scaffold after swelling, *ρ*_s_ is the density of the swelling agent (toluene), *ρ*_non__porous_ is the density of the nonporous PCLU network prepared in the same conditions compared to the porous scaffold without the addition of water, ρ_porous_ is the density of the porous PCLU scaffold and *P* is the scaffold porosity.

PCLU scaffold mechanical properties were determined by compression test using a universal machine. Scaffolds (18 mm in diameter, 8 mm in height) were tested during compression in the axial direction to the foam rise with a 500 N force range, 7 mm displacement range and 5 mm min^−1^ testing speed. The stress/strain relation was computer recorded and processed using Realview 3.0 software. The linear regression at the beginning of the stress/strain curve gave the effective modulus of elasticity *E*_1_* of the porous PCLU scaffold, while the linear regression at the end of the curve gave the elastic modulus of the corresponding nonporous material (*E*_non__porous_). The number average molecular weight between cross-links ${\bar{M}}_{c}$ of PCLU scaffolds was also determined using Equation (4) and compared to the value obtained through swelling experiments [28].

(4) $${\overset{-}{M}}_{c}=\frac{3\rho \mathrm{RT}}{E_{1}^{*}}$$

In Equation (4), *ρ* is the PCLU scaffold density (*ρ* = 159 kg m ^− 3^), *R* is the universal gas constant and *T* is the absolute temperature.

Mechanical properties were also evaluated when PCLU scaffolds were confined in 16 mm-diameter hole and the effective modulus of elasticity *E*_3_* of the confined porous PCLU scaffold was determined through linear regression at the beginning of the stress/strain curve.

The degradation assay was carried out according to ISO 10993-13 guideline standards. PCLU scaffolds were conditioned and sterilized as described below. After sterilization, 0.1 g of PCLU scaffolds were immersed in 1 ml of degradation medium composed of DMEM/Pen (100 IU ml^−1^)/Strep (100 µg ml^−1^)/Fz (2.5 µg ml^−1^), and incubated at 37°C for different periods of time. The pH of the degradation medium was monitored during the course of the study. At the end of each time point, scaffolds were removed from the medium and washed extensively in distilled water. Subsequently, scaffolds were air dried up to a constant mass. The mass loss was determined from the scaffold initial mass and their residual mass after drying. Mass and volumetric absorption ratios were determined from the mass of the wet scaffolds before drying and their residual mass after drying. Finally, PCLU scaffolds were characterized by FTIR-ATR analyses, ESEM, as well as through the determination of the porosity, WCA and number average molecular weight between cross-links.

The mass loss was also recorded during accelerated aging at 90°C. By taking an aging factor *Q*_10_ of 2–2.5 in accordance with ASTM F1980-16 standard, the rate of degradation is increased by a factor *f* ranging from 39.4 to 128.6 as calculated by Equation (5) [29]:

(5) $$f=Q_{10}^{\Delta T/10}$$

In Equation (5), Δ*T* is the difference between the elevated temperature used to accelerate the degradation process (90°C) and the temperature at which to study the effects of degradation (37°C). Therefore, the PCLU scaffold lifetime at 37°C was estimated to be that of the PCLU scaffold lifetime at 90°C multiplied by the factor *f*.

### Scaffold conditioning and sterilization

PCLU scaffolds were immersed in sterile water for 1 h under a vacuum system, followed by a 4-h immersion after water change. Thereafter, scaffolds were immersed for 1 h in 70 vol.% ethanol under a vacuum system. Finally, scaffolds were rinsed in sterile water overnight and autoclaved in wet condition.

### Fibroblasts colonization within PCLU scaffolds

For all *in vitro* studies, PCLU scaffolds were conditioned and sterilized as described above. Thereafter, scaffolds were incubated in DMEM/Pen (100 IU ml^−1^)/Strep (100 µg ml^−1^)/Fz (2.5 µg ml^−1^) for 12 h at 37°C in a humidiﬁed atmosphere of 5% CO_2_. The medium was removed before the use of the scaffold. Scaffolds were tested in triplicate in all experiments.

For static seeding experiments, PCLU scaffolds (3 mm in diameter, 2 mm in height) were placed in individual wells of a conical bottom 96-well plate, and 10 μl of a cell suspension at 5 × 10^7^ cells ml^−1^ in complete medium (DMEM/Pen (100 IU ml^−1^)/Strep (100 µg ml^−1^) supplemented with FBS (10%)) were seeded onto the scaffold, and incubated for 2 h at 37°C in a humidiﬁed atmosphere of 5% CO_2_. Then, each PCLU scaffold was carefully rinsed with PBS and incubated at 37°C for 10 min in a solution of trypsin (0.05%) and collagenase (0.025%). Thereafter, the supernatant was recovered and centrifuged, and the pellet of cells was resuspended. In order to take off all the cells from the PCLU scaffold, this operation is repeated three times. Then, the total number of cells was determined by counting with a Coulter Counter Z1 (Beckman) to assess cell adhesion efficiency onto the scaffolds.

For dynamic seeding experiments, two PCLU scaffolds (1 cm in diameter, 2 mm in height) were set on a needle immersed in a Corning^®^ mini bioreactor flooded with a cell suspension at 3 × 10^5^ cells ml^−1^ in complete medium, and equipped with a stirrer bar. The system was incubated for 2 h at 37°C in a humidiﬁed atmosphere of 5% CO_2_ under stirring at 350 rpm. Then, the scaffolds were carefully rinsed with PBS, and the cells were trypsinized and counted as described for the static seeding experiments. Some scaffolds were maintained up to 54 days in complete medium at 37°C in a humidiﬁed atmosphere of 5% CO_2_ under static conditions. Medium was changed once a week.

For migration experiments, fibroblasts at passage 5 resuspended in complete medium were seeded onto 24-well plates at 4 × 10^4^ cells per well. Fibroblasts were cultured at 37°C in a humidiﬁed atmosphere of 5% CO_2_ until an apparent 80% confluence was reached. Thereafter, one PCLU scaffold (1 cm in diameter, 2 mm in height) per well was carefully set down over the 80% confluent cell layer, and incubated in complete medium at 37°C in a humidiﬁed atmosphere of 5% CO_2_ up to 35 days. Medium was changed once a week. At 10, 25 and 35 days, the scaffolds were carefully rinsed with PBS, and the cells were detached and counted as described for static seeding experiments.

### hMSC proliferation and differentiation within PCLU scaffolds

PCLU scaffolds (1 cm in diameter, 2 mm in height) were conditioned and sterilized as described above. After sterilization, PCLU scaffolds were placed in individual wells of a 24-well plate and incubated in complete medium for 24 h at 37°C in a humidiﬁed atmosphere of 5% CO_2_. Thereafter, the medium was removed and 30 μl of a cell suspension at 5 × 10^6^ cells ml^−1^ were seeded on the scaffold and incubated for 3 h at 37°C in a humidiﬁed atmosphere of 5% CO_2_. Then, the wells were filled with 1 ml of either complete medium or osteogenic medium (complete medium containing 2-phospho-l-ascorbic acid and β-glycerophosphate) and incubated at 37°C in a humidiﬁed atmosphere of 5% CO_2_ up to 30 days. The medium was changed every 3 days. At 7, 14, 21 and 30 days, scaffolds were carefully rinsed with PBS. Scaffolds were tested in triplicate.

For cell counting, each PCLU scaffold was incubated at 37°C for 10 min in 2 ml of a trypsin (0.05%) and collagenase (0.025%) solution. Thereafter, the supernatant was collected and centrifuged, and the pellet of cells was resuspended. In order to recover all the cells from the PCLU scaffold, this operation is repeated three times. Then, the total number of cells was determined by counting with a Coulter Counter Z1 (Beckman).

Alizarin red staining was used to assess the MSC differentiation into mature osteoblasts. Scaffolds were immersed for 5 min in PBS/ethanol solution (50/50 vol.%), and then for 5 min in ethanol at −20°C. Thereafter, scaffolds were rinsed three times in distilled water, and cut in small pieces which were incubated in a 2% alizarin red solution. After 15 min, the solution was disposed and scaffolds were rinsed five times in distilled water. Afterwards, scaffolds were incubated in PBS for 15 min and the alizarin red fixed to the calcium-mineralized scaffold was detached by the action of a cetylpyridinium chloride solution (10%) for 15 min under stirring. The optical density of the recovered solution was read at 562 nm with a UVM 340 spectrophotometer.

For ESEM imaging, scaffolds were fixed in PFA (4%), rinsed in PBS and stored in ethanol 70 vol.% up to observation. Images were carried out using a Hitachi TM3000 ESEM operating at 5 kV and equipped with a Peltier stage operating at −4°C.

For DAPI staining, scaffolds were fixed in PFA (4%), rinsed in PBS and permeabilized with 1% triton X100 in PBS. Thereafter, scaffolds were immersed for 5 min in the dark in a DAPI solution (1/200). Scaffolds were then rinsed with PBS. Stained scaffolds were imaged on a Axioplan fluorescence microscope (Zeiss) connected to a Camedia CS550 color video camera (Olympus).

### Animal care and bone defect surgery

All animal treatments and procedures were approved by the Institutional Animal Care and Research Advisory Committee of IRBA in accordance with French law and international guidelines. Adult male Lewis rats (Janvier Labs) weighing 220–250 g were individually housed under standard temperature conditions (22°C ± 1°C) and hygrometry (45–60%) with a 12-h to 12-h light-dark cycle (lights on between 7 a.m. and 7 p.m.). Rats had free access to water and standard laboratory feed.

Sterilized PCLU scaffolds (3 mm in diameter, 2 mm in height) were incubated in complete medium 1 day before the bone defect surgery. All implantations were conducted under aseptic conditions.

At day 0, 18 rats were anesthetized by intraperitoneal injection of ketamine hydrochloride and medetomidine (60 mg kg^−1^ and 0.5 mg kg^−1^ of body weight, respectively). Skin incision and blunt dissection of the quadriceps were performed on the right shaved hind limb to expose the metaphyseal area of the distal femur. Defects were achieved by drilling a 3.0-mm diameter hole through the anterolateral cortical bone into the metaphyseal cancellous bone marrow, under continuous irrigation with saline. Scaffolds were carefully dried by absorption with a sterile compress at the time of implantation. Half rat population (*n* = 9; PCLU group) were implanted with PCLU scaffolds into osseous cavities, while the other half rat population remained nonimplanted (*n* = 9; control group). Muscles and skin were sutured (Vicryl^®^ 4/0) and rats were allowed to recover from anesthesia after an intramuscular injection of atipamezole (1 mg kg^−1^ of body weight). Analgesia was provided through subcutaneous injections of buprenorphine hydrochloride (50 µg kg^−1^ of body weight) 2 h after surgery and twice a day over three consecutive days. At 7, 15 and 30 days post-surgery, rats were sacrificed under sodium pentobarbital anesthesia (*n* = 3 rats per experimental time and group).

### Blood cell count

During rat sacrifice, blood was collected by intracardiac puncture with an EDTA-coated syringe (80 µl of 1.6% EDTA-K3 for every 800 µl blood samples) for blood cell count. A complete peripheral blood cell count was carried out using a Sysmex XNL-550 hematology analyser equipped with Fluorocell^®^ WDF (Sysmex reference N° AA-325-279) staining reagent kit and a rat-specific analysis software allowing blood cell sorting and counting.

### X-ray microcomputed tomography (micro-CT) analysis of rat femurs

After rat sacrifice, femurs were collected and fixed in Burkhardt’s solution [30] and scanned using a SkyScan 1174 microcomputed tomograph (SkyScan, Belgium) with the following parameter setups: source energy at 50 keV; intensity of 800 µA and isotropic voxel resolution of 15 µm with a 0.5 mm depth aluminum filter. All scans were 3D reconstructed and analysed with NRecon v.1.6 and CTan v.1.11 softwares (SkyScan), respectively, to separate mineralized structures from background using the software histogram tool to threshold gray-level values.

To assess bone repair efficiency in injured femurs, two regions of interest (ROI) were delineated by freehand drawing by the same investigator with a view to measure the closing of the cortical defect and the filling of the medullary cavity (lesion area) with trabecular new bone. The first ROI was a 3.0-mm diameter circle (equivalent to the drilled-hole diameter) positioned around the edges of the cortical defect. The surface of newly formed bone dedicated to the cortical defect closing was measured and compared with the surface of the initial hole defect (% of the cortical defect closing). The second ROI was bordered by the defect boundaries in the medullary cavity, encompassing regions containing newly mineralized bone and was defined as the BV/TV parameter (bone volume/tissue volume ratio).

### Histological examination of rat femurs

Following micro-CT imaging, the same sets of fixed femurs were dehydrated in methanol and processed for undecalcified histology in methylmethacrylate resin. Serial 6-µm thick longitudinal bone sections were prepared using a microtome equipped with a tungsten carbide blade (Leica 2055). Sections were stained with Masson–Goldner’s trichrome to identify bone structures, fibrous tissue and bone marrow cells. The PCLU scaffold was detected with a Sudan black B staining. Stained sections were imaged on a DMRB microscope (Leica) connected to a DXC930P color video camera (Sony).

### Data analysis

For measured parameters of PCLU scaffolds and for the *in vitro* and *in vivo* studies, values are expressed as mean ± standard deviation (SD).

## Results and discussion

### Characterization of PCLU scaffolds

**Figure 1:**
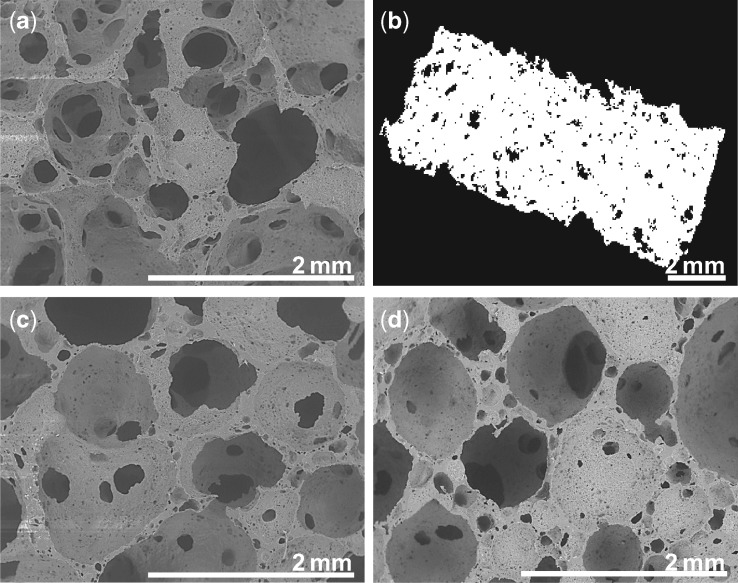
Porous structure of the PCLU scaffold: (a) ESEM image of the scaffold before conditioning and sterilization; (b) micro-CT image of the scaffold before conditioning and sterilization; (c) ESEM image of the scaffold after conditioning and sterilization; (d) ESEM image of the scaffold after 7 months of incubation at 37°C in the degradation medium

One major target in bone tissue engineering is to develop porous scaffolds with appropriate architecture since pore size, porosity and pore structure are crucial for scaffold osteoconductive properties [8]. Furthermore, it is of high importance to develop porous structure promoting cell spreading in three dimensions instead of a two-dimensional (2D) flattened spreading [31]. Finally, it is also required to develop an easy manufacturing process allowing the elaboration of scaffolds with various sizes and shapes in order to match the geometry of the bone defect. Recently, we have developed a poly(ester-urethane-urea) PCLU scaffold through poly(HIPE) elaboration that allows straightforward manufacturing and shaping [24]. Poly(HIPE) parameters were set up to obtain PCLU scaffolds that present pore size and porosity corresponding to the suggested features for bone tissue engineering [20]: a 85.1% porosity scaffold presenting highly interconnected pores without closed voids (Fig. 1b), and multiscale pore sizes ranging from 600 to 1800 μm for larger pore sizes, throat pore sizes as small as 150 μm and a fine porous morphology within the pore walls (pore size below 150 μm) (Fig. 1a). As determined through the micro-CT analysis, the thickness of the pore walls was found to be 104 ± 28 μm (Fig. 1b).

The mechanical behavior of the scaffold is also of importance, since cells regulate their metabolic activity and their response (shape, proliferation and cortical rigidity) depending on the scaffold rigidity and spatial structure [32, 33]. For instance, cortical stiffness and cell surface of stem cells have been shown to increase as the stiffness of the biomaterial increases from 1 to 20 kPa. Above 20 kPa, cell stiffness and surface are stable with values of 7 kPa and 6000 μm^2^, respectively [34]. As a consequence, elastomeric scaffolds developed with a modulus of elasticity higher than 40 kPa should be adequate to favor specific hMSC differentiation into osteogenesis lineage [33]. First, the PLCU scaffold mechanical behavior was determined through compression test in an uniaxial stress mode of compression where the scaffold only developed stresses in the compression direction and expanded freely in the two other directions. The effective modulus of elasticity *E*_1_* of the porous PCLU scaffold was found to be 161 ± 14 kPa and the number average molecular weight between cross-links ${\bar{M}}_{c}$ of the PCLU scaffold derived from *E*_1_* and calculated using Equation (4) (${\bar{M}}_{c}$ = 7340 g mol^−1^) was consistent with the value found by the swelling experiments (${\bar{M}}_{c}$ = 7930 ± 1600 g mol^−1^). Secondly, PCLU scaffold mechanical properties were studied when the scaffold was confined in a hole to mimic *in vivo* experiments. Indeed, PCLU scaffold is subjected to compression stresses in all 3D directions when implanted into bone defect cavity. As expected, compressing in triaxial stress mode of compression requires larger stresses than in uniaxial stress mode of compression. Therefore, the effective modulus of elasticity *E*_3_* was higher with a value of 321 kPa. Altogether, PCLU scaffolds always exhibited moduli of elasticity above 40 kPa and therefore seemed appropriate to induce hMSC differentiation into osteogenesis lineage.

### Stability of PCLU scaffolds

First of all, an important challenge in the development of polymeric scaffolds is to ensure effective sterilization of the biomaterial without causing drastic polymer degradation and modification of the porous structure. Regarding PU-based biomaterials, it is known that traditional and advanced sterilization methods can lead to polymer hydrolysis, oxidation and chain scission. Therefore, the sterilization process can elicit modifications of the biomaterial properties, and release of carcinogen 4,4′-methylenedianiline and analogs when PUs are synthesized with aromatic diisocyanate [35, 36]. Moreover in tissue engineering, scaffolds must degrade as the tissue regeneration occurs, without leading to cytotoxic degradation by-products. For bone regeneration, it has been suggested that scaffolds must possess reduced hydrophilicity so that the degradation rate may be longer than 18 months [37]. Indeed when using biodegradable poly(ester urethane) scaffolds in a femoral cortical defect model, the bridging of the cortical defect was not yet complete after 6 months of implantation [38]. A great care has to be given to degradation studies since it is well known that *in vivo* conditions are more severe than *in vitro* experiments leading to a decrease of the scaffold lifetime [39]. For instance, poly(ester urethane) scaffolds showed evidence of some degradation after 6 weeks of implantation in a femoral cortical defect model, while the mass loss reached only around 4% when the scaffolds were immersed in PBS solution at 37°C [38]. Furthermore, the structural parameters of the scaffold also influence the water permeability and therefore, the degradation behavior. For poly(ε-caprolactone)-based scaffolds, high porosity and pore size decrease hydrolysis and autocatalysis rate [20].

**Figure 2:**
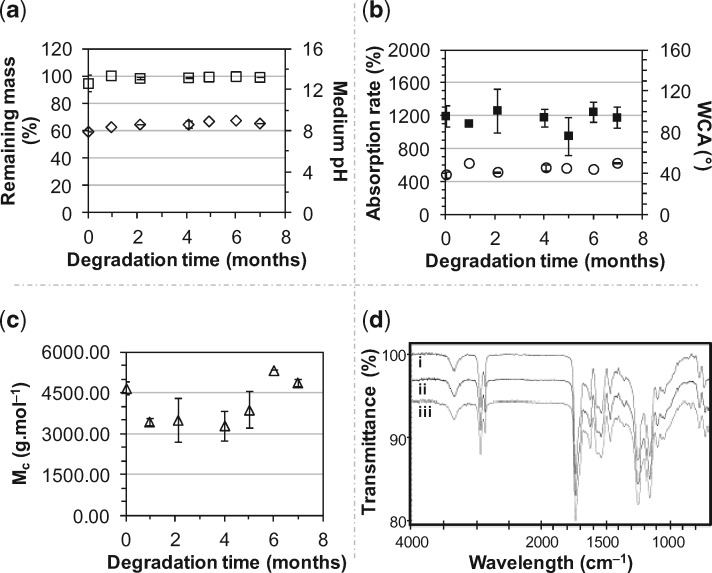
Incubation of PCLU scaffolds at 37°C in the degradation medium over a period of 7 months: (a) scaffold remaining mass (□) and medium pH (◊); (b) scaffold mass absorption rate (○) and WCA (■); (c) scaffold number average molecular weight between cross-links ${\bar{M}}_{c}$ (△); (d) FTIR-ATR spectra: (i) PCLU scaffold before conditioning and sterilization; (ii) PCLU scaffold after conditioning and sterilization; (iii) PCLU scaffold after 7 months of incubation at 37°C in the degradation medium

In our study, PCLU scaffolds were synthesized with (1) ε-caprolactone-based oligomers, which are known to be slowly hydrolyzed in low-concentrated caproic acid since it is rapidly metabolized, and (2) aliphatic diisocyanate, which is degraded in nontoxic amine [40, 41]. For the sterilization process, we developed a conditioning method that allows full distilled water uptake by the PCLU scaffold. Indeed after conditioning, the volumetric absorption ratio was found to be 106 ± 6%. After conditioning, PCLU scaffolds were sterilized in an autoclave under wet conditions. As shown in Fig. 1c and Table 1, the conditioning and sterilization process did not induce any modification of the porous structure and the porosity. No drastic degradation was detected since there was no change in the hydrophobicity (WCA—Table 1) and in the chemical composition of the biomaterial (FTIR—Fig. 2d and elemental composition—Table 1). However, a decrease in the number average molecular weight between cross-links was noticed (Table 1), which was in accordance with an increase in the elastic modulus (*E*_1_* after conditioning and sterilization = 243 kPa). Once again, there was a good correlation between the number average molecular weight between cross-links found by the swelling experiments (${\bar{M}}_{c}$ = 4670 ± 250 g mol^−1^) and the one derived from *E*_1_* and calculated from Equation (4) (${\bar{M}}_{c}$ = 4630 g mol^−1^). As expected, the effective modulus of elasticity was higher when the scaffold was confined in a hole during the compression test (*E*_3_* after conditioning and sterilization = 400 kPa).

**Table 1:** Porosity (*P*), WCA, number average molecular weight between cross-links ${\bar{M}}_{c}$^a^ and elemental composition of PCLU scaffolds before and after conditioning and sterilization

				Element (wt.%)	
	*P* (%)	WCA (°)	${\bar{M}}_{c}$ (g mol^–1^)^a^	Carbon	Oxygen
Before conditioning and sterilization	85.1±1.9	94±6	7930±1600	70.3±0.7	29.7±0.7
After conditioning and sterilization	85.8±1.7	95±10	4670±250	70.7±0.3	29.3±0.8

^a^ Determined through swelling experiments.

As the WCA was found to be around 95° (Table 1), PCLU scaffolds possessed hydrophobicity that seemed appropriate for the expected degradation rate for bone tissue engineering applications. The degradation study was carried out up to 7 months in DMEM medium at 37°C. PCLU scaffolds are sufficiently stable to be used in *in vivo* tissue engineering application for bone regeneration. Indeed, no significant chemical modification was noticed since scaffold mass (Fig. 2a), mass absorption rate and WCA (Fig. 2b) remained constant. After 7 months of incubation, the elemental composition of PCLU scaffold remained stable (%C = 71.2 ± 0.6 wt.%, %O = 28.8 ± 0.6 wt.%). No release of acidic degradation products was evidenced since there was no variation of the medium pH (Fig. 2a). However after 7 months of incubation, a very small variation of the PCLU backbone was detected by FTIR-ATR analysis through a decrease of 2.7% of the intensity of the peak at 1158 cm^−1^ corresponding to the stretching vibrations of the caprolactone ester group –COO–C– (Fig. 2d). This is in accordance with the small increase in the number average molecular weight between cross-links ${\bar{M}}_{c}$, which demonstrates the initiation of chain scissions (Fig. 2c). Regarding the porous structure, no variation of the porous morphology was noticed by ESEM analysis (Fig. 1d). The porosity and the volumetric absorption rate were stable with values of 85.0 ± 1.1% and 101.5 ± 0.5%, respectively, after 7 months of incubation. To estimate the over long timescale of scaffold degradation, accelerated aging was performed at 90°C. At this elevated temperature, it was noticed that mass loss started to occur after 15 days of incubation and the scaffold was very brittle. Using the factor *f* calculated by Equation (5), it was possible to conclude that the PCLU scaffold lifetime of 15 days at 90°C led to estimate the scaffold lifetime at 37°C in the range of 19.4–63.4 months. Taking into account that the lifetime is reduced *in vivo*, it seems that the PCLU degradation rate ranges in the appropriate timescale for bone regeneration. However, that point has to be confirmed by longer *in vivo* investigations than those performed in this study.

### Ability of PCLU scaffolds to be colonized by cells and to promote cells proliferation

Tissue engineering can be divided into two approaches. In the first cell-based approach, scaffolds are combined with cells in order to create *in vitro* a new engineered tissue substitute for further *in vivo* implantation; or seeded scaffolds are directly implanted and therefore act as carriers of cells to be delivered in the implantation site to enhance tissue regeneration. Some disadvantages of this approach are the heterogeneity of the tissues used as cell source, the extensive number of cells required to efficiently colonize the scaffold, the difficulty to ensure the homogeneity of the cell dispersion within the scaffold and the loss of cell proliferation potential due to culture expansion duration [42–45]. To overcome the problems encountered in cell-based tissue engineering, a second approach relies on the use of an inductive biomaterial capable of self-recruiting host cells from the site of injury to regenerate the tissue [42]. In both approaches, hydrophobicity, pore sizes, pore interconnectivity, and mechanical properties of the scaffold have a huge impact on the cell adhesion and distribution within the scaffold, the cell mobilization from host tissues, as well as cell-to-cell interactions [45–48].

**Figure 3:**
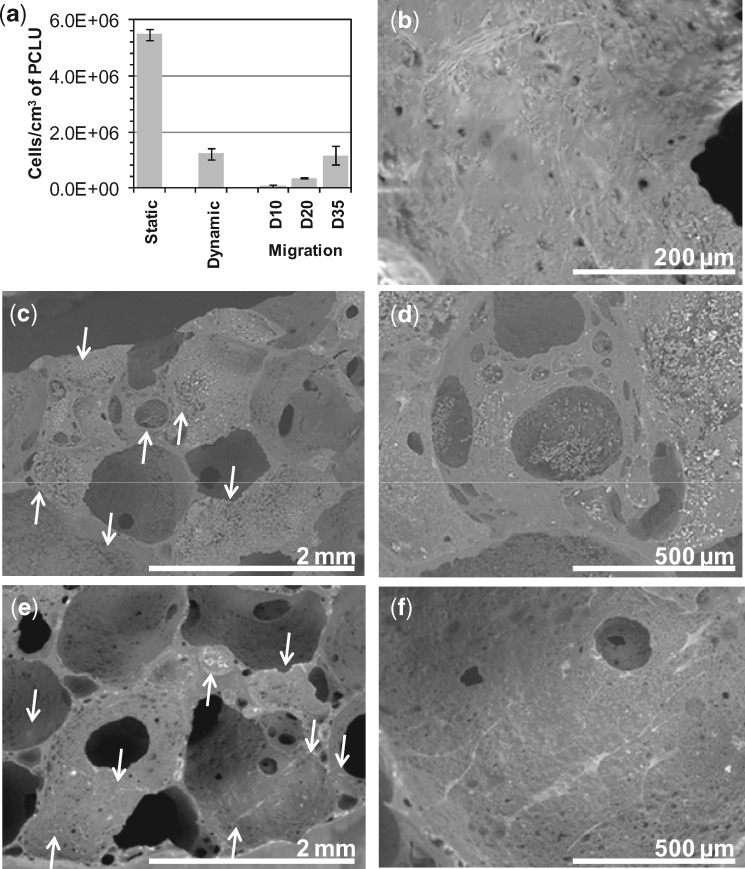
Cell colonization within PCLU scaffolds: (a) number of fibroblasts per cm^3^ of scaffold for direct static seeding (2 h), dynamic seeding (2 h) and migration at day 10, 20 and 35; (b) ESEM image of scaffold 54 days after the dynamic seeding; (c) and (d) ESEM images of scaffold after dynamic seeding (2 h); (e) and (f) ESEM images of scaffold after 35 days of fibroblast migration (arrows indicate some locations of cells in ESEM images)

As shown in Fig. 3a, we demonstrated the ability of PCLU scaffolds to allow cell infiltration and adhesion through direct seeding or by cell migration from outside to inside the scaffold. For the static seeding, small scaffold pieces were loaded with a high concentrated cell suspension to ensure that a large number of cells had a full access to the pore surface. As expected, a high number of cells (5.50 × 10^6^ ± 2.0 × 10^5^) adhered to the scaffold thanks to its moderate wettability, since fibroblasts have a maximum of adhesion on substrates having WCA around 60°–100° [46]. Fewer cells (1.24 × 10^6^ ± 2.0 × 10^5^) adhered on the scaffold with the dynamic seeding, but a less concentrated cell suspension was used and the scaffold bigger size limited the cell infiltration. The dynamic seeding highlighted that the scaffold structure was adequate for the penetration of the cell suspension, and therefore achieving a whole PCLU scaffold volume colonization with a spatially homogeneous distribution (Fig. 3c). Pore walls were covered with clusters of spherical cells after the seeding (Fig. 3d) and after 54 days of incubation, a thick layer of cells had surrounded the scaffold, and cells within the scaffold were spread over the pores indicating that the PCLU scaffold porosity and pore interconnectivity allowed cell survival (Fig. 3b). Regarding the migration skill of fibroblasts onto a biomaterial, it was reported that fibroblasts prefer migrating toward stiff surfaces [49]. It was also evidenced that fibroblasts have a reduced spreading but an increased motility on softer substrates [50, 51]. After 35 days of migration, we found that fibroblasts (1.17 × 10^6^ ± 3.5 × 10^5^) colonized the PCLU scaffold volume providing a reasonable proof that the mechanical signals and 3D environment of the soft scaffold were suitable enough for colonization by mobilized external cells (Fig. 3e). The cells were elongated on the pores surface and interacted to each other (Fig. 3f).

**Figure 4:**
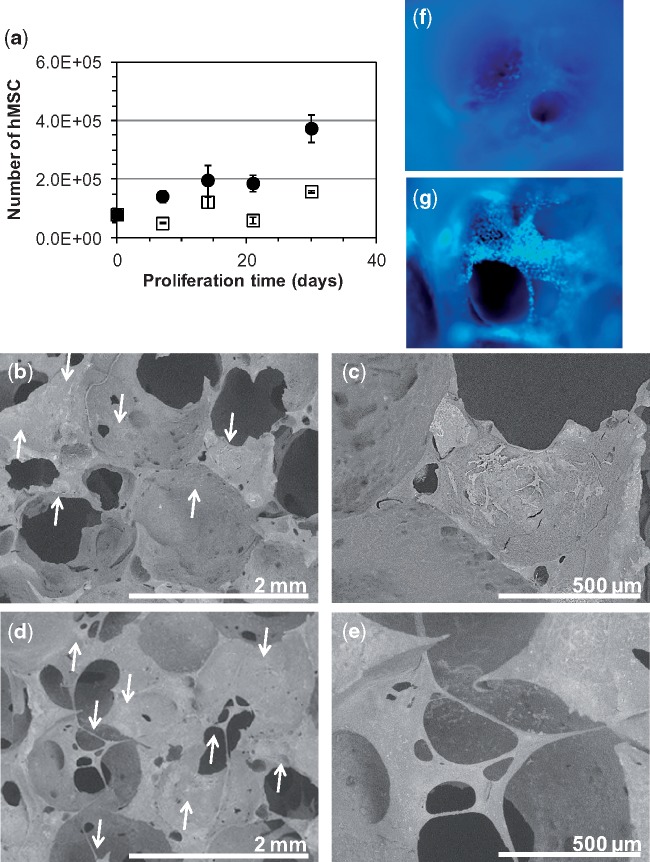
Cells development within PCLU scaffolds: (a) number of hMSC when cultured up to 30 days in complete medium (□) or osteogenic differentiation medium (●); (b) and (c) ESEM images of scaffold after 30 days of culture in complete medium; (d) and (e) ESEM images of scaffold after 30 days of culture in osteogenic differentiation medium; (f) DAPI staining of scaffold after 30 days of culture in complete medium—image of a pore (×50); (g) DAPI staining of scaffold after 30 days of culture in osteogenic differentiation medium—image of a pore (×50) (arrows indicate some locations of cells in ESEM images)

To validate that PCLU scaffolds were adequate for cell development, we studied the proliferation of hMSC within the scaffold when incubated in a complete medium or in an osteogenic differentiation medium. Then, hMSC were able to proliferate within the PCLU scaffold regardless of the medium; however a better proliferation (by nearly 2.4-fold at 30 days) was noticed when cells were cultured in the osteogenic differentiation medium which is similar to what is observed for a classical 2D culture onto tissue culture plate (Fig. 4a). In addition, hMSC were able to differentiate into osteogenic lineage (active osteoblasts) when cultured under appropriate induction, since the optical density of alizarin red staining was found to be increased by 9.3-fold after 30 days of culture in the osteogenic differentiation medium by comparison with the complete medium. Finally when developing scaffold for tissue engineering, it is of utmost importance that cells settle in the scaffold in a more natural morphological pattern, and interact on all sides with the pores surface. Interestingly, we found that cells spread over the surface of the pores in a 2D arrangement pattern when cultured in the complete medium (Fig. 4b, c and f), while they exhibited a 3D spatial distribution layout and filled the scaffold pores after 30 days of incubation in the osteogenic differentiation medium (Fig. 4d, e and g).

### PCLU scaffold ability to promote bone regeneration

Bone healing is a complex process involving release of cytokines, chemokines and growth factors; induction of signaling pathways with activation of thousands of genes; as well as progenitor cell proliferation and differentiation. All these mechanisms occur in a highly organized spatial and temporal process that eventually leads to bone regeneration resulting in an overall increase in volume of new skeletal tissues. Specific *in vivo* models can be used to evaluate the bone regeneration process, the bone–biomaterial interaction and pathophysiological evidence of ossification pathway modulation. In rat cavitary defect model, a 3-mm surgical cavity is drilled on the external face of the femoral distal head leading to a defect that does not heal without intervention. On the one hand, this model is able to produce bone morphogenetic factors, growth and differentiation factors that induce early bone repair mechanisms; on the other hand, chondrogenesis, osteogenesis and angiogenesis are affected. Therefore, the cavitary model is commonly used to investigate both the role and osteogenic properties of different biomaterials on bone healing [52–55].

**Figure 5:**
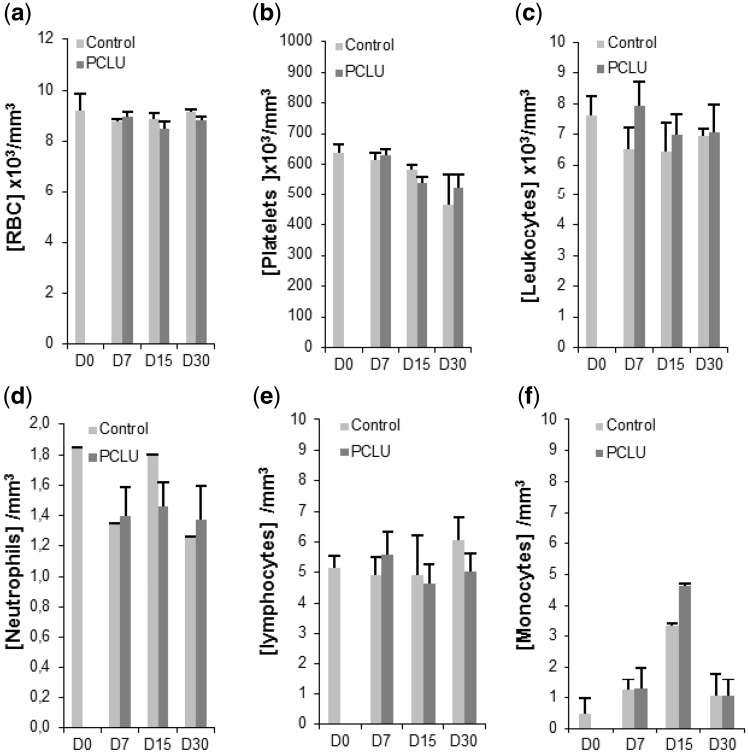
Blood analysis of the implanted rat group with the PCLU scaffold and the nonimplanted one up to 30 days after the drilling of the bone defect. Number of (a) red blood cells; (b) platelets; (c) leukocytes; (d) neutrophils; (e) lymphocytes; (f) monocytes (D0: rat blood analysis under anesthesia and before surgery. Data represent mean ± SD from the analysis of three animals per time)

First of all, *in vivo* experiments evidenced PCLU scaffold biocompatibility since neither complication, nor clinical sign of inflammatory response or scaffold rejection were noticed when scaffolds were implanted in cavitary defects up to 1 month. Indeed, the levels of circulating red blood cells and platelets were similar in the implanted rat group versus the nonimplanted one (Fig. 5a and b). In addition, no marked difference was found in the differential leukocyte count (Fig. 5c), and the same trend was observed in the variation of the numbers of circulating neutrophils or lymphocytes between the two groups (Fig. 5d and e). An increase in the circulating monocytes at day 15 (Fig. 5f) could reflect an improved bone remodeling since circulating monocytes are often recruited to sites of injury, and may differentiate into various cell types including osteoclasts, and have a critical role in the formation of new blood vessels [56]. Due to the nonbiological origin of the PCLU scaffold, a foreign body host response could be expected in the region of the biomaterial implantation. Surprisingly, such an immune response pattern was not evidenced as assessed by Masson–Goldner’s trichrome histological analysis. Whatever the considered post-implantation time, no typical foreign body giant cells (collection of fused macrophages usually generated in response to the presence of foreign body) was found in newly synthetized tissue located inside or in the close area all around the PCLU scaffold. Furthermore, no sign of PCLU fibrotic encapsulation was detected at late post-implantation time (day 30). Concerning the inflammatory response, some macrophages were predominantly located in the periphery of the lesion area at post-injury day 7 but PCLU implantation did not modified the qualitative aspect of this macrophage invasion due to the injury. However, more specific monocyte and macrophage immuno-staining performed on paraffin-embedded decalcified samples will be necessary to quantitatively validate these preliminary qualitative data.

**Figure 6:**
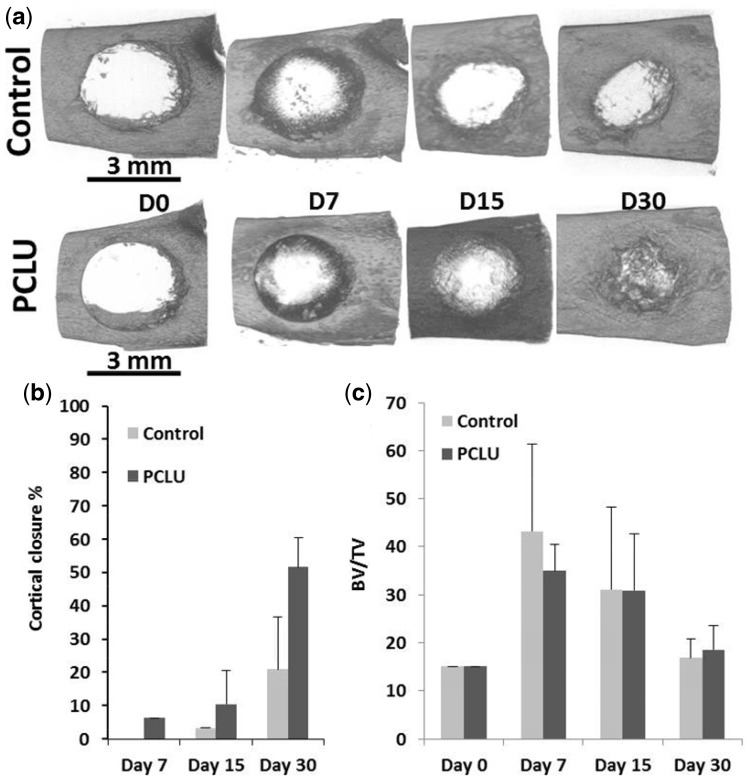
Micro-CT Analysis of the bone defect up to 30 days after surgery: (a) top view of the cortical defect; (b) cortical closure %; (c) bone volume/tissue volume ratio in the spongious cavity, representing trabecular bone (day 0 represents the trabecular bone content in normal intact bone. Data represent mean ± SD from the analysis of three animals per time)

The micro-CT analysis brought forward the osteoinductive ability of PCLU scaffolds. Although a natural repair of the bone defect was found in the nonimplanted rats group with a 20% defect closure at post-operative day 30, the closure efficiency increased by a 2.5 factor in the implanted rats group with the PCLU scaffold (Fig. 6a and b). In accordance with *in vitro* studies, this better *in vivo* bone regeneration may be explained by the enhancement of the recruitment of endogenous osteoblastic progenitor cells from the injured site, and their proliferation and differentiation throughout the PCLU scaffold microenvironment. The BV/TV of the defect area increased similarly during the first 2 weeks with or without PCLU scaffolds, and reached the intact bone BV/TV value by 30 days, reflecting the production and remodeling of new trabecular bone (Fig. 6c). However, in empty defects, bone healing remained mainly on the cavity edges up to 30 days.

**Figure 7:**
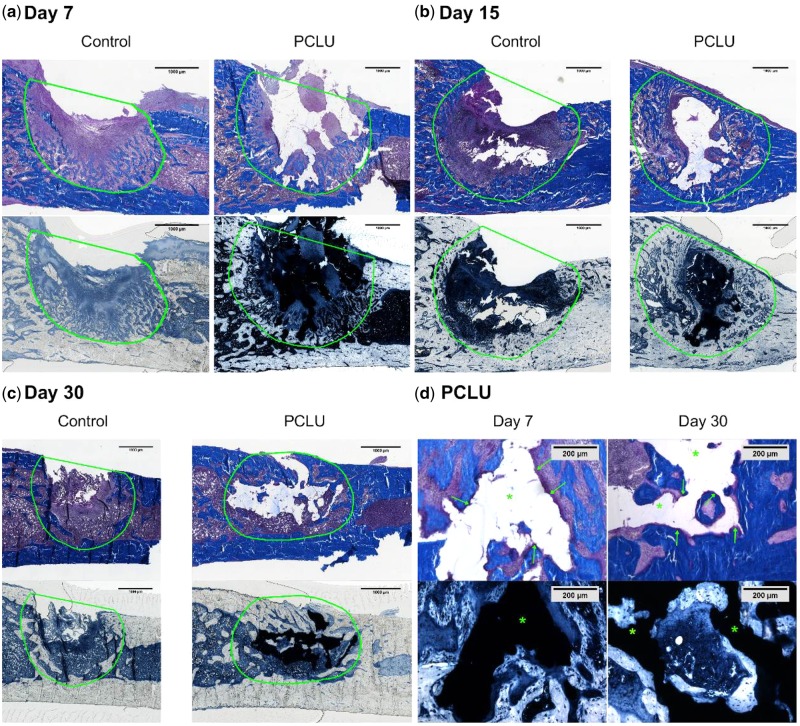
Histological analysis of the bone defect healing up to 30 days after bone surgery with or without PCLU scaffold implantation (a–b–c). Defect original location is indicated with green line. The bar represents 1 mm in (a–b–c) and 200 µm in (d). Masson’s trichrome stains mineralized bone in blue; Sudan black stains the PCLU scaffold in opaque black (arrows indicate active osteoid areas, asterisk indicates PCLU location)

The histological analysis clearly showed that the PCLU scaffold fit well in the bone defect defining an intimate contact between the bone defect borders and the scaffold, evidencing the easy fitting into the bone defect due to the biomaterial elasticity (Fig. 7d). The thickness of the remodeled bone on the defect borders increased throughout the 30 days of the study for the rat groups implanted with the PCLU scaffold (Fig. 7a–c), whereas this bone mineralization process stopped at 15 days and the bone defect remained empty at 30 days for the nonimplanted group (Fig. 7c). Moreover, osteoid areas were more numerous and extended as early as day 7 for the implanted rat group reflecting a higher number of active mature bone cells (Fig. 7d). It was also noticed that the PCLU scaffold was entrapped as early as day 15 by the newly formed bone trabeculae, and was totally split into lumps at day 30 (Fig. 7b and c). This newly formed bone in direct contact with the scaffold surface evidenced the osteoconductive properties of PCLU scaffolds. Finally, no fibrous tissue was detected up to 30 days of the PCLU scaffold implantation demonstrating its osteo-integration. No sign of cartilage was seen in the cavity of the defect, evidencing mainly intramembranous ossification for bone repair.

## Conclusions

We developed a chemically cross-linked ε-caprolactone-based poly(ester-urethane-urea) (PCLU) scaffold with a multiscale and interconnected porosity by a simple and controlled manufacturing process using an emulsion technique. *In vitro* experiments allowed concluding that PCLU scaffolds seemed to possess required osteoconductive properties for osteoprogenitor cell colonization and their further cell differentiation into mature osteoblasts. Thus, PCLU scaffolds seemed to act as 3D frameworks to guide tissue formation. Therefore, we highlighted that the PCLU scaffold, and more particularly its peculiar morphology and pore walls topography, provided a suitable environment for cell proliferation in a 3D spatial arrangement. *In vivo* experiments demonstrated the biocompatibility and the osteoconductive and osteoinductive properties of PCLU scaffolds resulting in a better bone reconstruction probably by the recruitment of osteoblastic progenitor cells, then their proliferation and finally their differentiation into mature osteoblasts. The bioactivity of PCLU scaffolds may be related to its structure, with micropores allowing fluid circulation, leading to degradation of the biomaterial, and macropores acting as a scaffold for bone cells, thus allowing bone growth. The scaffold *in vitro* stability and *in vivo* degradation during the 30 days study were sufficient for the tissue regeneration. Longer periods of investigation are currently underway. Overall, our study provided evidences of the PCLU scaffold capability to be used as a promising substitute for the treatment of bone defects.
